# Meta-Analysis of Exercise Effects on Cognition in Persons with Parkinson’s Disease

**DOI:** 10.3390/neurosci6020046

**Published:** 2025-05-23

**Authors:** Syed O. Ahmad, Dana Stiles, Kaylee Brown, Leah Dillon, Eliza Shroba

**Affiliations:** 1 Department of Occupational Science and Occupational Therapy, Neuroscience Core Faculty, Saint Louis University, St. Louis, MO 63103, USA; kaylee.brown@slu.edu (K.B.); leah.dillon@slu.edu (L.D.);; 2 Virginia Gore NeuroOccupation Lab, Inc., St. Louis, MO 63017, USA; 3 Office of Institutional Research, Saint Louis University, St. Louis, MO 63103, USA; dana.stiles@slu.edu

**Keywords:** occupational therapy, executive functioning, functional cognition, Parkinson’s disease, Alzheimer’s disease, meta-analysis, synthesis research

## Abstract

Background: Parkinson’s disease (PD) is a debilitating neurodegenerative disorder affecting millions of people worldwide. PD results in motor and cognitive dysfunction. While there is no proven cure for PD, it is widely agreed that aerobic exercises and occupations can help slow the progression of the disease and keep some motor-related symptoms from developing. The most effective forms of exercise to slow the progression of motor symptoms in Parkinson’s disease have also been studied. Research Question: This research article aims to compare the differences in outcomes of exercise on cognitive outcomes in Parkinson’s Disease, as evaluated by meta-analysis. Methods: Key terms were Parkinson’s Disease and exercise terms. These search terms were then entered to electronic databases—Ovid MEDLINE, SCOPUS, and CINAHL—from March 2018 to May 2023. An ancestral bibliography was also performed. Results: Two reviewers screened the title and abstract records (*n* = 528) found in the initial search. Our review identified 18 studies which met inclusion criteria for meta-analysis. The meta-analysis found an effect of exercise on cognition of patients with PD (d = −0.03) which was not significant (CI95% of −0.13 < µ < 0.08; *p* > 0.05, as the CI includes zero). Additionally, the homogeneity analysis was not significant (Q (17) = 2.83; *p* > 0.05).

## 1. Introduction

Parkinson’s disease (PD) is a debilitating progressive neurodegenerative disorder affecting millions of people worldwide. PD results in both cognitive and motor dysfunction, featuring symptoms such as bradykinesia, postural instability, muscular rigidity, speech disturbances, dystonia, sleep disturbances, pain, and anxiety [1,2,3,4]. It has been long known that exercise has benefits for the motor deficits in PD, and this database was used to find a positive result utilizing Unified Parkinson’s Disease Rating Scale (UPDRS) motor scores [1]. Exercise appears to enhance neuroplasticity by increasing the production of brain-derived neurotrophic factor (BDNF), a protein linked to improved learning, memory, and resilience against neurodegeneration [5,6,7,8,9]. Studies show that aerobic exercise, in particular, improves brain function and delays cognitive decline by promoting blood flow to the brain, enhancing oxygen and nutrient delivery to regions like the hippocampus, which is crucial for memory [7].

Exercise and cognition have been studied in the past for Parkinson’s disease, and some of the outcomes have been positive [10], looking at treadmill activities and the initiation and severity of symptoms. Recent meta-analyses have been performed on factors associated with sedentary activity and progression [11], which found improvement in non-motor scores, including for cognition. A recent systematic review of seven studies found negative cognitive outcomes from sedentary activities on cognition in PD [12]. Non-motor effects of non-pharmaceutical therapies were meta-analyzed to find positive results (cognition among several non-motor symptoms) [13]. This conclusion was supported by research which found that the pattern and style of exercise did not show differences in PD symptomatology, but resulted in overall improvement [10].

The effects of exercise on cognition and exercise have been variable. In a multi-disciplinary study of intensive rehabilitation, cognitive improvements were noted, though not isolated to the exercise portion, and additional non-reported cognitive interventions were utilized [9] Basdisarre). Insignificant correlations between exercise, cognitive training, and subcortical disease were found in a recent systematic review [14]. A systematic review that reached farther back in time found inconclusive and confounding results for exercise, cognition, and PD [15]. This justifies a further look into the phenomenon.

This database was chosen for its range of activities and its focus on diagnosed PD (excluding other basal brain motor dysfunction), and was used to compare endurance-based activities with occupation-based activities to show there were identical motor improvements between the groups [2,3]. Motor outcomes for this dataset were previously reported, and this analysis took the initial set of papers and further examined the Montreal Cognitive Assessment (MoCA) and Trailmaking A outcomes in the same PD groups [1]. MoCA, The Montreal Cognitive Assessment (MoCA), is a validated cognitive screening tool that can be used to screen for CI. It is a brief, 30-item questionnaire that assesses different cognitive domains, including executive function, language, orientation, memory, and visuospatial abilities [16]. It is designed to be a quick and easy-to-administer assessment tool that can be completed in approximately 10 min by trained personnel. The MoCA has also been validated in various clinical settings against neuropsychological batteries, demonstrating reliability and validity in detecting Mild Cognitive Impairment (MCI) and dementia [16,17].

Parkinson’s disease and Alzheimer’s disease often co-occur [18]. Several genetic variations have been identified for both PD and AD, but the underlying mechanisms of the gene profile are largely unknown [19]. The likelihood of an individual developing PD or AD results from the interaction between genetic and nongenetic factors over the person’s lifetime, and demonstrates statistically related impairment [19]. PD and AD often co-occur, highlighting a complex relationship between their pathologies. PD primarily affects motor function due to dopamine neuron degeneration in the substantia nigra and related cognitive impairments, and the proteopathies include Lewy body cellular inclusions [20]. The profile of disability differs in AD, and affects memory and cognitive abilities; the proteopathies include beta-amyloid plaques and tau neurofibrillary tangles in the brain [21]. Studies suggest overlapping mechanisms, such as protein misfolding, mitochondrial dysfunction, neuroinflammation, and oxidative stress, may contribute to co-occurrence [21]. In spite of the differences in pathology, cognitive and executive functioning impairments being common to both diseases, rehabilitative approaches and treatment have been similar for both disorders, which makes rehabilitative approach an area that requires more exploration.

Problem Statement: This research article aims to compare the differences in outcomes of exercise on cognitive outcomes in Parkinson’s disease, as evaluated by meta-analysis.

**Hypothesis** **1:** *Exercise interventions will have positive cognitive results for PD*, *similar to positive results seen for AD*. *Null hypothesis*: *exercise does not significantly affect cognitive decline in people with PD*.

## 2. Materials and Methods

Multiple a priori criteria were used to determine the inclusion and exclusion of the studies considered for our meta-analysis. All studies had to examine the effect of activity on cognition-related outcomes of Parkinson’s Disease. For cognition-related outcomes, data were measured by the MoCA or Trail Making Test Part A to be considered for inclusion. For the purpose of this analysis, physical intervention included activities such as dance, yoga, treadmill training, archery, water sports, and aerobic exercise, as well as many others. In order to be selected for meta-analysis, all studies were reviewed and selected for inclusion if they meet the following criteria.

1. Included individuals with a Parkinson’s disease diagnosis.
2. Performed assessment using Trail Making Tests Part A, or the MoCA.
3. Excluded dual-task interventions.
4. Used a physical activity for treatment or intervention.
5. Written in the English language, and full text available.
6. Published in a peer-reviewed journal.
7. Published between 2018 and October 2023 (these are the dates the database spans).

Studies were excluded if they did not meet all of the inclusion criteria or if they studied only animals.

### 2.1. Literature Search

Our review of the literature began with the formulation and selection of key search terms. These terms were Parkinson’s Disease and exercise terms (e.g., dancing, aerobic exercise, endurance exercise, yoga, swimming, and boxing). These search terms were then entered simultaneously to search for literature across three electronic databases: Ovid MEDLINE, SCOPUS, and CINAHL. These databases were systematically searched in October 2023. An ancestral bibliography was also performed using selected bibliographies.

### 2.2. Review Process

**Figure 1:**
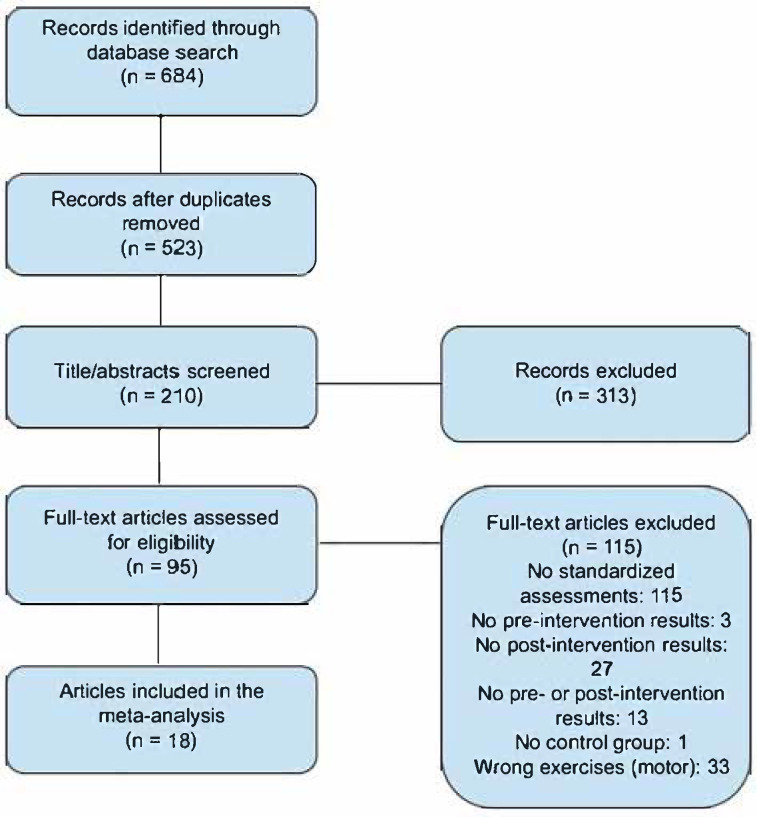
PRISMA flow diagram.

The search yielded 684 citations based on our inclusion criteria of being written in the English language, of studies being performed on humans, and of being specific studies (clinical trials, randomized trials). After duplicates were removed, a total of 523 articles remained. Two researchers screened the titles and abstracts of the articles, ruling out 313 records, with 210 remaining for further assessment. The remaining articles had their full-text assessed for eligibility based on the inclusion of results of tests including the Unified Parkinson’s Disease Rating Scale, Montreal Cognitive Assessment, or the Trail Making Test A. Of the articles reviewed, 115 did not include any of the included standardized assessments, leaving 95 articles. Additionally, 3 were excluded due to not having pre-intervention test results, 27 for not having post-intervention test results, 13 for not having pre- or post-intervention results, and 1 was excluded due to not having a control group consisting of individuals with Parkinson’s disease. Furthermore, 33 were excluded as they focused on motor exercises instead of cognitive exercises. With this, 18 articles were selected for inclusion into the study [see Table 1]. The summary of the review process can be found in the PRISMA chart (see Figure 1).

### 2.3. Meta-Analysis Calculation Formulae


$$d_{i}=\frac{\chi_{1}-\chi_{2}}{\left(\frac{{SD}_{1}+{SD}_{2}}{2}\right)}$$

$$w_{i}=\frac{\left(2\left(n_{i1}+n_{i2}\right)n_{i1}n_{i2}\right)}{2{\left(n_{i1}+n_{i2}\right)}^{2}+n_{i1}n_{i2}{d_{i}}^{2}}$$

$$d=\frac{\sum d_{i}w_{i}}{\sum w_{i}}$$

$$95\%CI=d_{i}\;\pm 1.96\;\sqrt{\frac{1}{\sum w_{i}}}$$

$$Q=\sum {d_{i}}^{2}w_{i}-\frac{{\left(\sum w_{i}d_{i}\right)}^{2}}{\sum w_{i}}$$

$$I^{2}=(Q-df)/Q\;*\;100\%$$

## 3. Results

**Figure 2:**
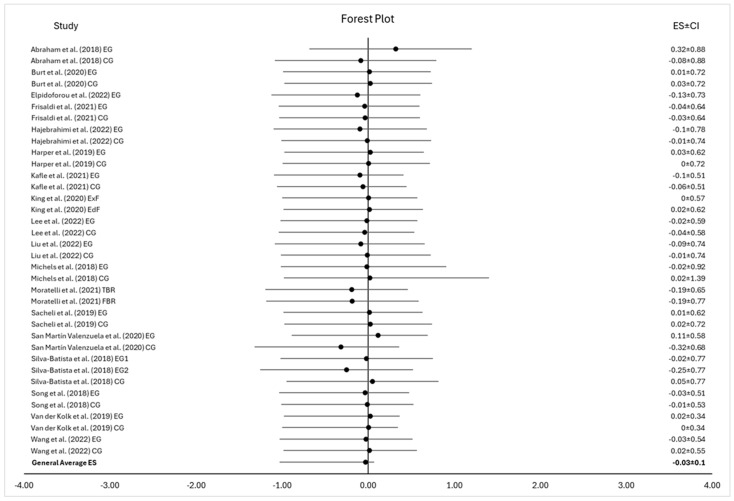
The forest plot for the meta-analysis of cognitive outcomes from all studies. Note. • = study effect size, ―•― = the black lines represent the Confidence Intervals (CI) of a study, ES ± CI represents the effect size and the 95% confidence interval of the study. EG = Experimental Group, CG = Control Group, ExF = exercise first group, EdF = education first group, TBR = two-beat rhythm, FBR = four-beat rhythm [22,23,24,25,26,27,28,29,30,31,32,33,34,35,36,37,38,39].

Our review identified 18 studies which met inclusion criteria for meta-analysis. The meta-analysis, which focused only on exercise’s effects on cognition, found a negative overall effect of exercise on cognition in patients with PD (d = −0.03), and was not significant (CI95% of −0.13 < µ < 0.08; *p* > 0.05, as the CI includes zero) [see Table 2 and Figure 2]. Intervals that include zero are non-conclusive and non-significant. Additionally, the homogeneity analysis was not significant (Q (18) = 2.83, *p* > 0.05). Further, the heterogeneity analysis, I^2^ = −501%, was negative and thus equivalent to 0% heterogeneity, suggesting that all variability in the study effect-size estimates is due to sampling error, and, therefore, no observed heterogeneity [40,41]. Thus, we accept the null hypothesis, that exercise does not significantly affect cognitive decline in people with PD.

## 4. Conclusions

With the acceptance of the null hypothesis, it begs the following question: why does exercise improve cognition in AD, but not in PD? Regular physical activity, particularly aerobic and resistance training, have been shown to significantly enhance neuroplasticity, which refers to the brain’s ability to adapt, reorganize, and form new neural connections associated with aging conditions [42]. Exercise has been found to improve executive brain function. This includes key cognitive processes like problem-solving, planning, and multitasking, which have been shown to slow the progression of cognitive decline often associated with PD and AD [43]. Alzheimer’s disease (AD) and Parkinson’s disease (PD) are also characterized by abnormal protein aggregation and progressive neuronal loss [44]. AD is associated with amyloid-beta plaques and neurofibrillary tangles composed of hyperphosphorylated tau protein affecting the hippocampus and cerebral cortex, leading to memory loss and cognitive decline [44]. PD involves α-synuclein aggregates forming Lewy bodies in the substantia nigra, causing dopaminergic neuronal loss and motor symptoms [45]. Growing evidence shows that Alzheimer’s disease-related pathologies such as tau and amyloid beta also play a role in PD, especially for cognitive impairment in PD [46]. Despite these distinct pathologies, both diseases often impact overlapping brain regions, such as the hippocampus and cortex, in later stages of PD, and the basal forebrain, in AD. The overlapping mechanisms complicate the diagnosis and treatment but underscore the need for integrated research to better understand shared pathways and develop therapies addressing both conditions (AD and PD).

It has been seen, in vitro, that the role of inflammation in neurodegenerative disorders with genetics plays an important role in this relationship [47]. Significant correlations have been observed between the levels of cerebrospinal fluid tau and A-beta with cognition status, as well as between Apolipoprotein E and microtubule-associated protein tau (MAPT) genotypes in PD [46]. Dysregulation of cholesterol metabolism has been implicated in the pathogenesis of AD and PD [44]. With the use of phytosterols, improvements have been seen in reducing cholesterol levels [44]. Other studies have shown that phytosterols have an anti-inflammatory and antioxidant effect, changing the pathologies of AD and PD [44]. Studies suggest that physical activity stimulates the production of brain-derived neurotrophic factor (BDNF), a protein that plays a vital role in supporting the growth, survival, and repair of neurons [6,7,8]. Elevated levels of BDNF, coupled with improved cerebral blood flow from exercise, enhance the health of brain regions critical for memory, attention, and decision-making, such as the hippocampus and prefrontal cortex [6,7,8]. Research indicates that regular physical exercise has significant benefits for cognition in individuals with Alzheimer’s disease (AD) [5]. Additionally, exercise helps reduce neuroinflammation and oxidative stress, both of which contribute to Alzheimer’s pathology [6]. Research suggests that consistent moderate-intensity physical activity can slow the progression of cognitive symptoms in AD. Though applied similarly, this study indicates that the mechanism for cognitive decline differs significantly in PD than AD, though the functional cognitive limitations are similar. This requires therapists and researchers to seek activities that more directly address the cognitive decline in PD, so results start to match those in AD.

In addition to aerobic and resistance exercises, structured programs like tai chi, dance, and yoga offer unique cognitive and motor benefits [2]. This database was used to compare the two approaches, and found significant differences in motor outcomes, regardless of the approach [2]. These activities not only improve balance, flexibility, and coordination—key motor challenges in PD—but also incorporate elements of rhythm, concentration, and learning of new movement patterns, providing mental stimulation—key cognitive challenges in both AD and PD [1,42]. Dance, for instance, combines physical activity with music, which has been shown to engage multiple brain areas simultaneously, fostering both motor and cognitive improvements. Similarly, tai chi emphasizes slow, deliberate movements and mindfulness, which can enhance focus and reduce stress, further benefiting cognitive health. Yoga promotes relaxation, breathing control, and body awareness, which contribute to overall brain health. The meta-analysis results are surprising, given the similarity of cognitive deficits and the inability to move the needle in PD, as opposed to positive results in AD across the literature [2]. Key occupational and physical therapy interventions use practice models, such as the neuro-occupation model, which design rehabilitation treatment approaches based on mechanisms of neuroplasticity that drive cognitive changes through performing exercises and activity. Exercise and brain adaptability is crucial for maintaining cognitive function and countering the effects of neurodegeneration in degenerative conditions, such as AD and PD. However, clinically, rehabilitation follows similar cognitive retraining and activities for both diseases, assuming similar mechanisms of disease etiology and progression.

The key takeaways from this meta-analysis are as follows: first, regardless of activity (endurance-based or occupation-based), exercise has positive effects for motor scores in PD, but not in cognitive scores. Second, more research needs to be performed on the mechanistic overlap between PD and AD, in order to develop more effective rehabilitative strategies for the treatment of clients with PD.

The limitations of this meta-analysis are, firstly, that the results encompass 2018 to October 2023, so publications with relevant results may have been published before or after this time; however, the results are robust for the time period. Future meta-analyses can update the results, which will strengthen any results derived from the process. Second, many of the studies subjected to meta-analysis in previous studies include motor results, but lack reporting of cognitive outcomes using standardized tests (such as MoCA and Trail Making Test A). We hope more PD exercise studies include cognitive evaluation, which would also strengthen already-robust results.

## Figures and Tables

**Table 1:** Characteristics of studies included in meta-analysis.

Author	Exercise Group	Group	Description	PD Outcome	Test Interval	Trail A/MoCA Pre-Test Mean (SD)	Pre-Test Group Size (ni1)	Trail A/MoCA Post-Test Mean (SD)	Post-Test Group Size (ni2)
Abraham et al. (2018) [22]	Dynamic Neuro-Cognitive Imagery	EG	2 h x day	Trail A	2 weeks	30.55 (10.19)	10	27.68 (7.06)	10
		DNI	2 h x day			27.46 (10.33)	10	28.18 (7.03)	10
Elpidoforou et al. (2022) [23]	Dance	EG	60 min 2x/wk	MoCA	8 weeks	23.92 (1.47)	16	27.15 (0.75)	13
Liu et al. (2022) [24]		EG	60 min 2x/wk		8 weeks	24.7 (3.7)	14	26.9 (2.3)	14
		CG	60 min 2x/wk			26.1 (3.8)	14	26.4 (4.1)	14
Moratelli et al. (2021) [25]		Binary dance	45 min 2x/wk	MoCA	12 weeks	19.5 (5.33)	18	23.66 (4.51)	18
		Quaternary dance	45 min 2x/wk			17.3 (6.35)	13	20.84 (6.55)	13
Michels et al. (2018) [26]		EG	60 min 1x/wk	MoCA	10 weeks	27 (2.18)	9	27.44 (2.4)	9
		CG	60 min 1x/wk			25.25 (1.5)	4	24.75 (0.96)	4
Frisaldi et al. (2021) [27]		EG	120 min 3x/wk	MoCA	5 weeks	26.08 (3.07)	19	27.11 (2.51)	19
		CG	60 min 3x/wk		5 weeks	25.68 (2.89)	19	26.55 (2.62)	19
Lee et al. (2022) [28]	Treadmill Training	EG	60 min 2x/wk	MoCA	8 weeks	26.36 (0.66)	22	26.82 (0.64)	22
		CG	60 min 2x/wk			26.35 (0.69)	23	27.39 (0.59)	23
Burt et al. (2020) [29]		EG	minimum of 15 min 3x/wk	MoCA	12 weeks	25.33 (4.12)	15	25 (2.33)	15
		CG	minimum of 15 min 3x/wk			26.2 (2.46)	15	25.53 (2.61)	15
San Martín Valenzuela et al. (2020) [30]		EG	60 min 2x wk	Trial A	10 weeks	51.3 (28.1)	23	48.86 (14.91)	23
		CG	60 min 2x wk			58.74 (45.46)	17	72.73 (42.65)	17
Hajebrahimi et al. (2022) [31]	Virtual Reality	EG	60 min 3x/wk	MoCA	4 weeks	22.27 (2.19)	15	24.54 (1.5)	11
		CG	60 min 3x/wk			22.76 (3.39)	15	23 (4.91)	13
Wang et al. (2022) [32]		Wu Qin Xi	90 min 3x/wk	MoCA	24 weeks	26.7 (1.55)	30	27.43 (2.11)	23
		Stretching	90 min 3x/wk			27.5 (1.77)	30	27.05 (2.22)	22
Kafle et al. (2021) [33]	Aerobic	EG	60 min 2x/wk	MoCA	7 weeks	26.4 (4.88)	30	29.07 (4.54)	30
		CG	60 min 2x/wk			26.27 (4.28)	30	27.93 (4.4)	30
Sacheli et al. (2019) [34]		EG	40–60 min 3x/wk	MoCA	3 months	27.94 (1.98)	20	27.53 (1.94)	20
		CG	40–60 min 3x/wk			28.23 (1.48)	15	27.54 (2.57)	15
Van der Kolk et al. (2019) [35]		EG	30–45 min 3x/wk	MoCA	6 months	26.3 (2.2)	65	25.7 (0.5)	65
		CG	30–45 min 3x/wk			26 (6.3)	65	25.9 (0.5)	65
Song et al. (2018) [36]		EG	min 15 min 3x/wk	MoCA	12 weeks	26.4 (2.77)	31	27.3 (2.8)	28
		CG	min 15 min 3x/wk			26.5 (2.7)	29	26.7 (2.3)	25
Harper et al. (2019) [37]	Cycling	EG	40 min 3x/wk	MoCA	1 week	25.7 (2.8)	20	25 (3.2)	20
		CG	N/A			25.7 (3.2)	15	25.6 (3.3)	15
King et al. (2020) [38]	Agility Boot Camp	Exercise First	80 min 3x/wk	MoCA	6 weeks	26.6 (3)	25	26.5 (3.1)	23
		Education First	240 min /wk			24.3 (4.2)	21	23.9 (4.3)	19
Silva-Batista et al. (2018) [39]	Resistance Training	RT	60 min 2x/wk	MoCA	12 weeks	21.8 (4.3)	13	22.2 (3)	13
		RT w/instability	60 min 2x/wk			20.8 (3.2)	13	26.8 (2.4)	13
		CG	n/a			22.7 (5.7)	13	21.6 (6.5)	13

Note. Group refers to participant grouping type (EG = Experimental Group; CG = Control Group, DNI = Did Not Include). Description provides a brief explanation of the activity/exercise conducted in the study by each group. PD outcome refers to the cognitive measurement reported in the study. Test interval refers to how long the study/intervention was conducted/data were collected. Pre-test total *n* = 756; post-test total *n* = 721.

**Table 2:** Summary of effect size calculations for studies included in meta-analysis.

Study	Exercise Type	Group	d*_i_*	w*_i_*	d*_i_*^2^w*_i_*	d*_i_*w*_i_*	1.96 x SE	LCI	UCI
Abraham et al. (2018) [22]	Dynamic Neuro-Cognitive Imagery	EG	0.32	4.94	0.51	1.58	0.88	1.20	3.21
		DNI	−0.08	5.00	0.03	−0.41	0.88	−0.96	3.28
Elpidoforou et al. (2022) [23]	Dance	EG	−0.13	7.16	0.11	−0.91	0.73	−0.86	5.72
Liu et al. (2022) [24]		EG	−0.09	6.99	0.05	−0.60	0.74	−0.83	5.54
		CG	−0.01	7.00	0.00	−0.08	0.74	−0.75	5.55
Moratelli et al. (2021) [25]		Binary dance	−0.19	8.96	0.33	−1.73	0.65	−0.85	7.67
		Quaternary dance	−0.19	6.47	0.22	−1.20	0.77	−0.96	4.96
Michels et al. (2018) [26]		EG	−0.02	4.50	0.00	−0.07	0.92	−0.94	2.69
		CG	0.02	2.00	0.00	0.04	1.39	−1.37	−0.72
Frisaldi et al. (2021) [27]		EG	−0.04	9.50	0.01	−0.37	0.64	−0.67	8.25
		CG	−0.03	9.50	0.01	−0.32	0.64	−0.67	8.25
Lee et al. (2022) [28]	Treadmill Training	EG	−0.02	11.00	0.00	−0.19	0.59	−0.61	9.84
		CG	−0.04	11.50	0.02	−0.45	0.58	−0.62	10.36
Burt et al. (2020) [29]		EG	0.01	7.50	0.00	0.10	0.72	−0.70	6.10
		CG	0.03	7.50	0.01	0.19	0.72	−0.69	6.10
San Martín Valenzuela et al. (2020) [30]		EG	0.11	11.48	0.15	1.30	0.58	−0.46	10.35
		CG	−0.32	8.39	0.85	−2.67	0.68	−0.99	7.07
Hajebrahimi et al. (2022) [31]	Virtual Reality	EG	−0.10	6.34	0.06	−0.61	0.78	−0.88	4.81
		CG	−0.01	6.96	0.00	−0.07	0.74	−0.75	5.51
Wang et al. (2022) [32]		Wu Qin Xi	−0.03	13.02	0.01	−0.35	0.54	−0.57	11.95
		Stretching	0.02	12.69	0.00	0.21	0.55	−0.53	11.61
Kafle et al. (2021) [33]	Aerobic	EG	−0.10	14.98	0.14	−1.44	0.51	−0.60	13.99
		CG	−0.06	14.99	0.06	−0.92	0.51	−0.57	14.00
Sacheli et al. (2019) [34]		EG	0.01	10.00	0.00	0.15	0.62	−0.61	8.78
		CG	0.02	7.50	0.00	0.19	0.72	−0.69	6.10
Van der Kolk et al. (2019) [35]		EG	0.02	32.50	0.02	0.75	0.34	−0.32	31.82
		CG	0.00	32.50	0.00	0.13	0.34	−0.34	31.83
Song et al. (2018) [36]		EG	−0.03	14.71	0.02	−0.49	0.51	−0.54	13.71
		CG	−0.01	13.43	0.00	−0.10	0.53	−0.54	12.38
Harper et al. (2019) [37]	Cycling	EG	0.03	10.00	0.01	0.28	0.62	−0.59	8.78
		CG	0.00	7.50	0.00	0.03	0.72	−0.71	6.10
King et al. (2020) [38]	Agility Boot Camp	Exercise First	0.00	11.98	0.00	0.05	0.57	−0.56	10.87
		Education First	0.02	9.97	0.00	0.17	0.62	−0.60	8.76
Silva-Batista et al. (2018) [39]	Resistance Training	RT	−0.02	6.50	0.00	−0.12	0.77	−0.79	4.99
		RT w/instability	−0.25	6.45	0.41	−1.63	0.77	−1.02	4.94
		CG	0.05	6.50	0.02	0.32	0.77	−0.72	4.99
**∑**			−1.07	367.90	3.06	−9.25	24.37	−23.67	320.15
d*_i_* = −0.03 CI_d95%_ = −0.03 ± 0.1 *Q* = 2.83									

Note. d*_i_* = unweighted effect size, w*_i_* = weighted effect size, EG = Experimental Group, CG = Control Group, DNI = Did Not Include, d = d-index, average weighted effect size for all studies, CI_d95%_ = 95% confidence interval for the average weighted d-index, *Q* = q-statistic, homogeneity analysis.

## Data Availability

Data are contained within the article and all original articles are available via Ovid MEDLINE, SCOPUS, and CINAHL for the period from March 2018 to May 2023. Further inquiries can be directed to the corresponding author, S.A.

## Abbreviations

The following abbreviations are used in this manuscript:

PD	Parkinson’s Disease
AD	Alzheimer’s Disease
BDNF	Brain-derived Neurotrophic Factor
UPDRS	Unified Parkinson’s Disease Rating Scale
MoCA	Montreal Cognitive Assessment
MAPT	Microtubule-Associated Protein Tau
CI	Confidence Interval
